# Heterogenous Expression and Purification of Lipid II Flippase from *Staphylococcus aureus*

**DOI:** 10.2174/0109298665316374240531113258

**Published:** 2024-07-04

**Authors:** Yuan Yuan Zheng, Wai-Hong Chung, Yun-Chung Leung, Kwok-Yin Wong

**Affiliations:** 1 State Key Laboratory of Chemical Biology and Drug Discovery, Department of Applied Biology and Chemical Technology, The Hong Kong Polytechnic University, Hung Hom, Hong Kong, China

**Keywords:** *Staphylococcus aureus*, lipid II Flippase, MurJ, protein purification, detergent screening, buffer

## Abstract

**Background:**

*Staphylococcus aureus* is a common pathogen with strains that are resistant to existing antibiotics. MurJ from *S. aureus* (SaMurJ), an integral membrane protein functioning as Lipid II flippase, is a potential target for developing new antibacterial agents against this pathogen. Successful expression and purification of this protein shall be useful in the development of drugs against this target.

**Objective:**

In this study, we demonstrated the optimized expression and purification procedures of SaMurJ, identified suitable detergent for extracting and solubilizing the protein, and examined the peptidisc system to generate a detergent-free environment.

**Methods:**

SaMurJ fused with N-terminal ten-His tag was expressed without induction. Six detergents were selected for screening the most efficient candidate for extraction and solubilization of the protein. The thermostability of the detergent-solubilized protein was assessed by evaluated temperature incubation. Different ratios of peptidisc bi-helical peptide (NSP_r_) to SaMurJ were mixed and the on-bead peptidisc assembly method was applied.

**Results:**

SaMurJ expressed in BL21(DE3) was confirmed by peptide fingerprinting, with a yield of 1 mg SaMurJ per liter culture. DDM was identified as the optimum detergent for solubilization and the nickel affinity column enabled SaMurJ purification with a purity of ~88%. However, NSP_r_ could not stabilize SaMurJ.

**Conclusion:**

The expression and purification of SaMurJ were successful, with high purity and good yield. SaMurJ can be solubilized and stabilized by a DDM-containing buffer.

## 1 INTRODUCTION

The emergence of antimicrobial resistance (AMR) has become one of the major threats to public health and the global economy [1-3]. Centers for Disease Control and Prevention estimated an annual loss of approximately 35 billion dollars in productivity in the United States due to AMR-related expenses [4]. In 2021, The World Health Organization (WHO) also declared that AMR is one of the top 10 global public health threats [5]. As more clinical isolates showed multiple or extended resistance to existing antibiotics, the identification of novel drug targets is urgently needed for developing new antibiotics.

MurJ, a key component of peptidoglycan biosynthesis [6-9], is a promising target owing to its essentiality in cyto kinesis and cell integrity in many bacteria. This integral membrane protein belongs to the multidrug/oligo-saccharidyl-lipid/polysaccharide (MOP) flippase superfamily [6-10], which is responsible for transporting various substrates across the cytoplasmic membrane. MurJ and its homologs translocate the peptidoglycan precursor, lipid II molecules, from the inner to the outer leaflet of the membrane, where it is polymerized and cross-linked by glycosyltransferase and transpeptidase [11, 12]. This step is critical for peptidoglycan synthesis as knockout or inactivation of this protein results in lethality or hypersensitivity to β-lactam antibiotics in several bacteria, including *E. coli* [13], *B. cenocepacia* [14], *B. pseudomallei* [15], and *S. aureus* [16, 17].

For structure-based drug development, a detailed understanding of the structure, dynamics, and interaction with the substrate/inhibitor of this flippase is essential [18-20]. Sham *et al.* showed that MurJ is the lipid II flippase by using a thiol-specific labeling agent to selectively inhibit MurJ mutants [21], which is one of the most convincing works. Bolla *et al.* further confirmed that MurJ forms a stable complex with lipid II by using native mass spectrometry, whereas no stable lipid II - FtsW complex can be observed, to argue against FtsW being the lipid II flippase [22]. The first crystal structures of MurJ from *T. africanus* were solved at a resolution of 2.0-Å [10, 23]. The lipid II binding pose was predicted by *in silico* docking based on the crystal structure. The structure of *E. coli* MurJ in two conformations was also resolved by Kohga *et al.* [24] and Zheng *et al.* [25], providing more information on the action and folding of this flippase. In the same paper by Kohga *et al.* [24], the structures of two states of the *Arsenophonus endosymbiont* ortholog were reported.

*Staphylococcus aureus* is a formidable pathogen, and many clinically isolated strains exhibit extensive or multi-drug resistance [26-29]. The structure of the MurJ from this bacteria is valuable for antibiotic development. However, all structures of MurJ up to this date were from (three) Gram-negative bacteria. Structure prediction is of limited use as the homology is quite low and *S. aureus* is Gram-positive. For instance, the sequence identity between the *T. africanus* and *S. aureus* orthologues is only 19.3%. There were a few studies related to identifying inhibitors of MurJ of *S. aureus in vivo* (hereafter denoted as SaMurJ). It is worth pointing out that 3 compounds have already been discovered and synthesized that inhibit the SaMurJ [16, 17], but the detailed interaction between these compounds and this protein was not understood. Although the inhibitory efficiencies of these compounds can be optimized by random modifications coupled with high-throughput screening, structure-based rational design is highly desirable to provide an alternative route to lead optimization. It is in this context that we undertake to develop a method for obtaining high-purity SaMurJ samples for structural analysis and inhibitor development.

## 2 MATERIALS AND METHODS

### 2.1 Bacterial Strain, Plasmid, and Construct

The gene of the MurJ homolog in *S. aureus* denoted as SaMurJ, was codon-optimized for *E. coli* expression. Additional nucleotides encoded 25 amino acid residues (MGSSHHHHHHHHHHSQDAENLYFQG) were added to the 5’ end of the gene, which was synthesized and cloned into pET28a between the NcoI and XhoI sites and named as pET28a- SaMurJ. On expression, it produces a protein with a ten-histidine tag added at its N-terminus, followed by a TEV protease site. *E. coli* strain BL21(DE3) was selected as the host for the expression.

### 2.2 Detergent Screening

To identify suitable detergents for solubilization, SaMurJ was expressed in 2 liters 2xYT medium supplemented with 50 µg/ml kanamycin. The cell pellet from a 2 L culture was resuspended in buffer A (50 mM HEPES pH 7.6, 200 mM NaCl and 10% glycerol), lysed by sonication with DNase (20 µg/ml) and the cOmplete^TM^ Protease Inhibitor Cocktail (Roche) added, and clarified by centrifuge at 10,000 g. The membrane fragment containing supernatant was then aliquoted into 6 equal portions for ultra-centrifugation at 200,000 g for 1 hour at 4°C to collect the membrane fraction. The pelleted membrane fraction in each aliquot was resuspended in buffer A supplemented with one of the following detergents: (1) 1% Brij L23 (BJ), (2) 1% sucrose -6-monolaurate (SML), (3) 1% dodecyl-β-maltoside (DDM), (4) 1% decyl-β-maltoside (DM), (5) 1% octyl-β-glucoside (OG), or (6) 1% lauryldimethylamine oxide (LDAO). The mixture was stirred at 4°C for 2 hours to completely resuspend the total membrane (TM) fraction. After collection of 40 μl samples for SDS-PAGE and Western blotting, each TM resuspension was ultra-centrifuged at 200,000 g at 4°C again to remove the insoluble materials. The supernatant was denoted as the soluble membrane (SM) fraction. Together with the TM fraction resuspended by different detergents, they were analyzed by SDS-PAGE and Western blotting. Western blotting was done by using a primary rabbit antibody against His-tag (Cell Signaling Technology Inc, catalog number 2365) and an anti-rabbit antibody (Thermofisher Scientific, catalog number 65-6120). All image acquisition was conducted by a Bio-Rad ChemiDoc system.

### 2.3 Protein Expression at Various Temperatures

BL21(DE3) cells transformed with the plasmids were plated on LB agar supplemented with 50 µg/ml kanamycin. A single colony was inoculated into a 20 ml pre-culture in 2xYT medium supplemented with kanamycin at a final concentration of 50 µg/ml and grown under agitation at 250 rpm overnight at 37°C. A shaker flask containing 500 ml of 2xYT broth was inoculated with 5 ml of the pre-culture, followed by incubation at 37°C and agitation at 250 rpm until the optical density at 600 nm (OD_600_) reached 0.8-1.0. The flasks were then transferred to a bath at 16, 20, or 37°C respectively and agitated at 250 rpm for a further 20 hours without induction. Cell was harvested by centrifugation at 8,000 g for 20 min and the pellet was stored at -80°C before cell disruption and purification.

Cell pellets from the 500 ml culture were resuspended in buffer A, lysed and clarified as described above. All the membrane fraction pellet was resuspended in buffer A supplemented with 1% dodecyl-β-maltoside (DDM). The purification of the DDM solubilized protein solution was conducted on a Bio-rad NGC Quest chromatography system connected with a 1 ml HisTrap FF Crude column (Cytiva) charged with Nickel. After loading the sample, the column was washed with 10 - 15 ml buffer A with 0.05% DDM until the absorbance at 280 nm of the eluent was lower than 100 mAU, followed by gradient (0 - 50 mM for 10 ml) and step (100 mM for 10 ml) imidazole wash. The target protein was eluted at 500 mM imidazole and was analyzed by SDS-PAGE and UV-spectrophotometry. The protein identity was confirmed by peptide mass fingerprinting of SDS-PAGE-resolved samples (section 2.6).

### 2.4 Comparison of DDM and SML Solubilized SaMurJ

BL21(DE3) transformed with pET28a- SaMurJ was cultured and lysed as described in section 2.3, except the culture size became 2L. The cell was harvested and aliquoted into two equal portions for DDM and SML solubilization. The protein was purified as described in section 2.3 and the stability of the two samples was compared by using evaluated temperature incubation. Samples solubilized in DDM or SML were diluted to 0.5 mg/ml and were incubated at various temperatures from 30 - 80°C for 30 min, followed by 10 min incubation on ice and centrifugation at 15,000 g for 5 min to completely remove all precipitates. The supernatant was analyzed using SDS-PAGE and size exclusion chromatography. Size exclusion chromatography was conducted by an Agilent 1290 UHPLC system and ACQUITY UPLC Protein BEH SEC Column, 200 Å, 1.7 µm, 4.6 mm X 150 mm and equilibrated with buffer A containing 0.05% DDM or SML. The SEC column has also been checked using the reference standards from Waters (BEH200 SEC Protein Standard Mix-Asia), and the retention volumes of the standard proteins (thyroglobulin, immunoglobulin G, myoglobin, and uracil) were noted in the chromatograms.

### 2.5 Peptidisc Assembly

The reported 37 amino-acid residues amphipathic bi-helical peptide (denoted as NSP_r_) [30-33] with sequence FAEKFKEAVKDYFAKFWDPAAEKLKEAVKDYFAKLWD was synthesized by Synpeptide Co Ltd. A 5 mg/ml NSP_r_ stock solution was prepared in buffer A. In this experiment, the reported assembly procedure was adopted [30]: 200 μl purified SaMurJ solubilized in DDM (0.5 mg/ml) was directly mixed with 200 μl buffer A containing 30-, 60-, and 90-fold molar excess NSP_r_, and incubated at 4°C overnight. A control sample was prepared by adding 200 μl buffer A without NSP_r_. Before 10-fold dilution with buffer A, a 10 μl sample was collected from the assembly mixture for SDS-PAGE analysis, followed by loading onto 200 μl nickel affinity resin. The resin was washed with 2 ml buffer A, and the SaMurJ-NSP_r_ complex was eluted by 200 μl buffer A supplemented with 500 mM imidazole. The eluted solution was analyzed by SDS-PAGE.

### 2.6 In-gel Digestion and Peptide Mass Fingerprinting

The identity of the purified sample was confirmed by peptide mapping [34]. In brief, the target band on the SDS-PAGE gel of the purified SaMurJ was excised and washed with an acetonitrile and water mixture, followed by overnight trypsin or pepsin digestion at 37°C. The protein digest was purified by a C18 desalting spin column (ThermoFisher) and analyzed by the Agilent 6540 UPLC-MS system.

### 2.7 Protein Quantification

The concentration of SaMurJ in solution was estimated by measuring its absorbance at 280 nm and converting with the molar extinction coefficient of 82,174 M^-1^cm^-1^. The relative protein quantity on SDS-PAGE was evaluated by ImageJ [35].

## 3 RESULTS AND DISCUSSION

### 3.1 Detergent Screening

The selection of suitable detergent for target protein solubilization is the first critical step in studying membrane protein [36, 37]. In this study, six non-ionic detergents (BJ, SML, DDM, DM, OG, and LDAO) were screened and analyzed using SDS-PAGE and Western blotting. Due to the significant amount of background host cell protein, the target protein could not be easily identified in the SDS-PAGE. However, we can still observe an intense band slightly lower than the 52 kDa protein marker. The intensity of the band in different lanes varied significantly with the detergent used. Using Western blotting with anti-His antibody to detect the recombinant SaMurJ provided a reasonably good level of selectivity to the protein, as well as a better quantification and solubilization estimation. By measuring the intensity ratio of the target band of soluble membrane fraction (SM) to total membrane (TM) fraction, the solubilization efficiency can be compared.

**Fig. (1):**
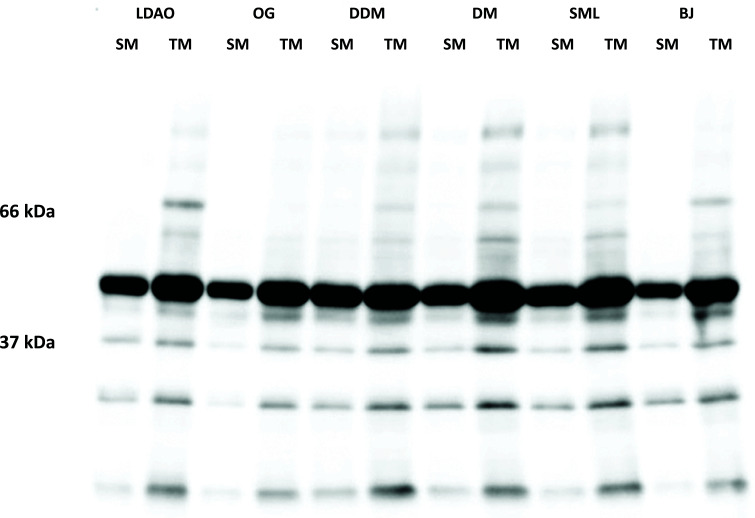
Membrane fractions were solubilized in 6 different detergents and analyzed by Western blotting. To evaluate the solubilization efficiency, the intensity ratio of SM:TM in the Westen blot for each detergent was analyzed by ImageJ. The SM:TM ratio of the detergents are 54.7% (LDAO), 49.9% (OG), 80.9% (DDM), 47.7% (DM), 58.4% (SML), and 32.8% (BJ).

We found that DDM was the most effective solubilizing agent. About 80% of SaMurJ in the total membrane fraction can be solubilized. The second best detergent, SML, gives a fairly good solubilization with 58%, whereas with BJ, only 33% of SaMurJ can be extracted from the total membrane fraction (Fig. **1**). DM, OG, and LDAO showed similar solubilization capability, with SM:TM ratio ranging from55 - 48%. However, we observed that precipitates formed in the DM, OG, and LDAO-solubilized membrane fractions within a short period (data not shown), which could block the column during the chromatographic purification. Therefore BJ, DM, OG, and LDAO were not selected for SaMurJ solubilization in the following experiments.

### 3.2 Protein Expression and Purification

**Fig. (2):**
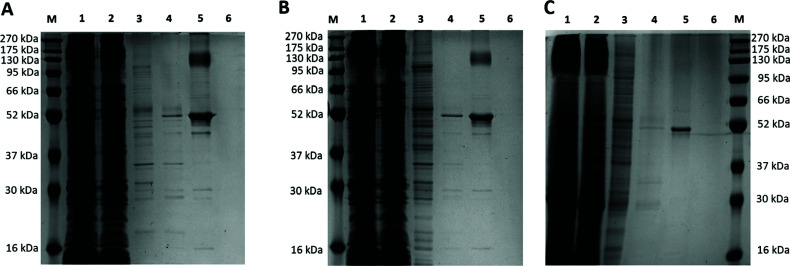
Expression of SaMurJ at different temperatures without induction. SaMurJ was expressed in BL21(DE3) with overnight incubation at (**A**) 16°C, (**B**) 20°C, and (**C**) 37°C. Lane M: protein ladder, lane 1: membrane fraction before column, lane 2: Flow through, lane 3: washed with 5 mM imidazole, lane 4: washed with 100mM imidazole, lane 5: elution of target protein at 500 mM imidazole, lane 6: washed with 1 M imidazole. The amount of protein purified from 16°C, 20°C, and 37°C culture were 0.48 mg, 0.35 mg, and 0.17 mg respectively.

Membrane proteins are usually difficult to express as it is in general, unstable and toxic to the host when overexpressed [38, 39]. Optimization of the expression condition is usually required to avoid protein aggregation. In this study, we adopted a simple approach to over-produce membrane protein in BL21(DE3) cells in 2xYT medium, with overnight incubation without IPTG induction [40]. Although this strategy did not provide precise control of the expression level, Zhang *et al.* reported that the expression of five out of eight selected membrane proteins in the “leaky” BL21(DE3) was superior to those from C41(DE3) and C43(DE3) with IPTG induction [40]. We found this induction-free strategy successful for SaMurJ, and the expression level was highly dependent on the incubation temperature (Fig. **2**). With 500 ml culture, the amount of SaMurJ obtained from expression at 16°C was 0.48 mg (Fig. **2A**), which was about three folds higher than that at 37°C (0.17 mg) (Figs. **2B**, **C**).

**Fig. (3):**
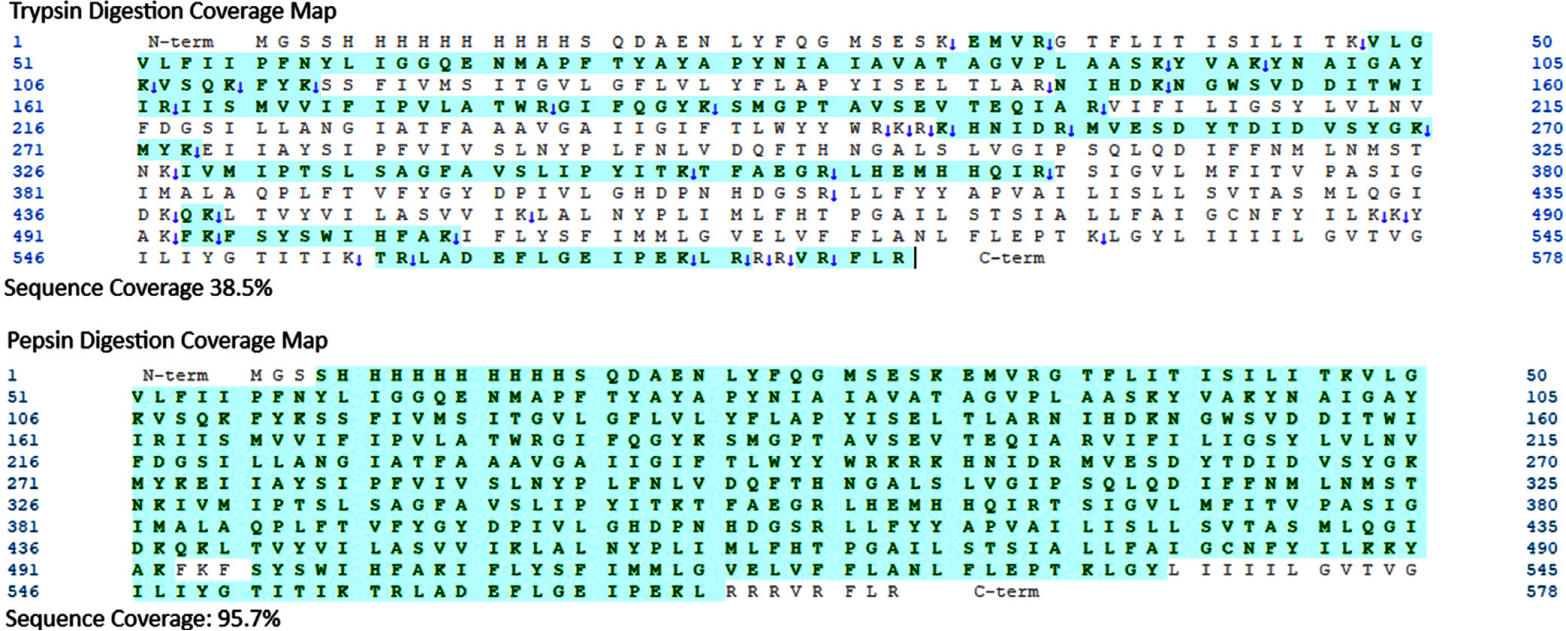
Peptide Mass Fingerprinting Coverage Map. The identity of the purified SaMurJ was confirmed by trypsin and pepsin in-gel digestion. The highlights in green color represent the covered sequences. Relatively low coverage (38.5%) was obtained by trypsin digestion. Pepsin digestion provided a higher coverage (95.7%) confirming that the purified protein was not truncated nor auto-digested. The detail information of the identified peptides are listed in Tables ST1 and ST2 in the Supplementary Material.

From the SDS-PAGE of the purified SaMurJ, the appearance molecular weight was about 52 kDa, which is much smaller than the theoretical molecular weight (64.9 kDa). To confirm the purified protein was full-length SaMurJ, the target band on SDS-PAGE was cut for in-gel digestion, followed by mass spectrometry peptide mass fingerprinting analysis. Trypsin was at first used for digestion, but the sequence coverage was relatively low (Fig. **3**). Indeed, low sequence coverage is quite common when membrane protein is being digested by trypsin with a significant number of large and very hydrophobic peptide fragments in the digest [41-43]. We then used pepsin for digestion, obtaining sequence coverage higher than 95% (Fig. **3**), with 124 peptides.

SaMurJ sequence was identified, which allowed us to ensure that the purified SaMurJ was not truncated nor auto-digested. Such discrimination between the theoretical and appearance molecular weight on SDS-PAGE would most probably result from the “Gel shifting” effect, commonly observed in SDS PAGE of membrane proteins [44].

### 3.3 Comparing SML and DDM Solubilized SaMurJ

**Fig. (4):**
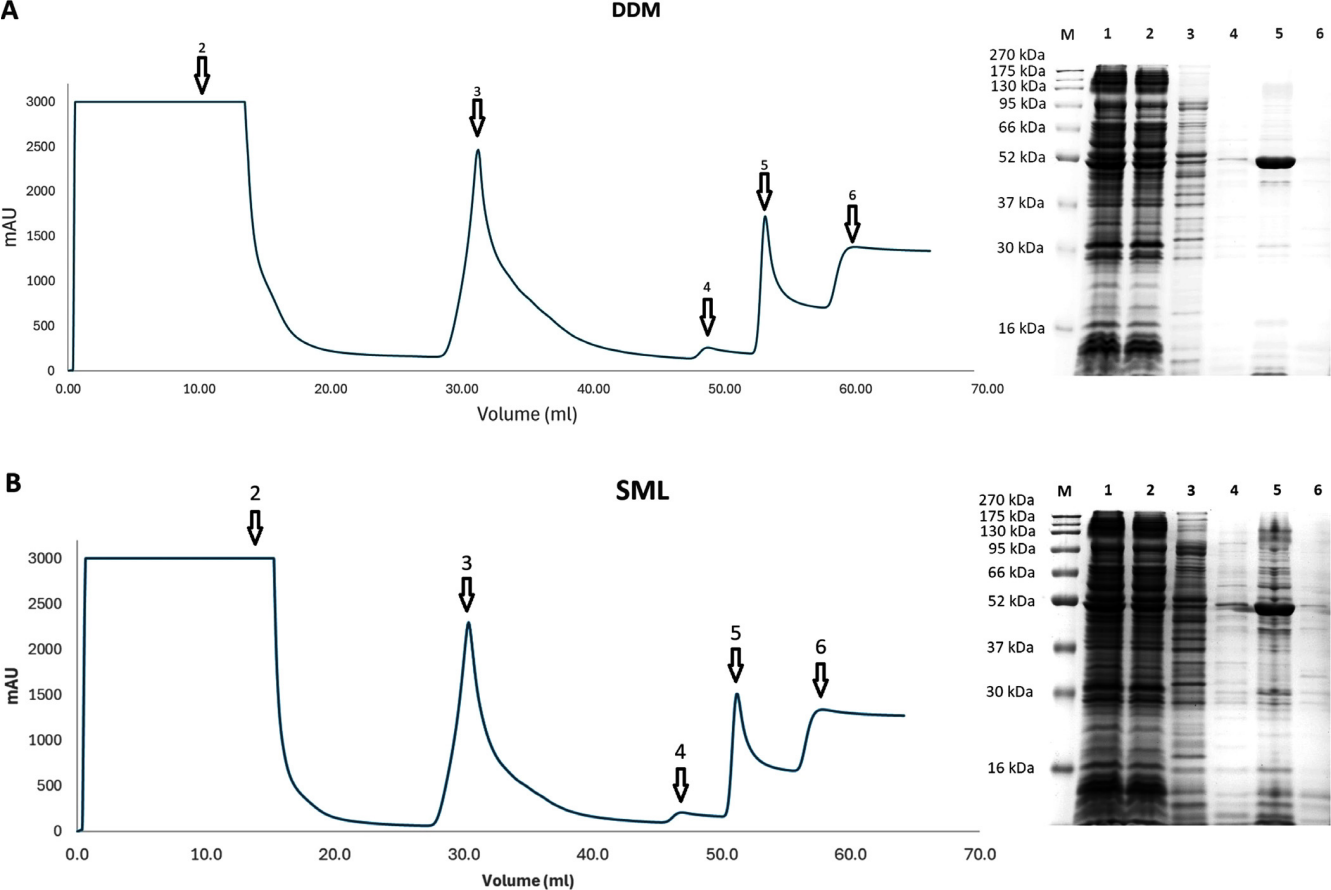
Purification of DDM and SML solubilized SaMurJ. Membrane fractions solubilized in (**A**) 1% DDM or (**B**) 1% SML were loaded onto a 1 ml HisTrap Crude column. The protein concentrations of the 2 ml-target fractions (lanes 5) were 1.0 mg/ml (DDM) and 0.8 mg/ml (SML) respectively. Lane 1: membrane fraction before column, lane 2: flow through, lane 3: washed with 5 mM imidazole, lane 4: washed with 100 mM imidazole, lane 5: elution at 500 mM imidazole, lane 6: washed with 1 M imidazole.

Among the detergents screened in this study, only DDM and SML can solubilize SaMurJ without precipitate formation. In addition, SML has physical and chemical properties similar to DDM (Tables **S1**-**S3**) when compared with other detergents. We were curious whether SML could stabilize SaMurJ, although the solubilization efficiency was slightly better than that of the other detergents used. Indeed, we found that the purification profile and yield were similar, with 1.0 mg (DDM) and 0.8 mg (SML) SaMurJ obtained from 1 L culture (Fig. **4**). However, the purity of SML solubilized SaMurJ was significantly lower than DDM solubilized SaMurJ (75% *vs*. 88%). It was likely that SML solubilized much more other host proteins in the membrane fraction.

**Fig. (5):**
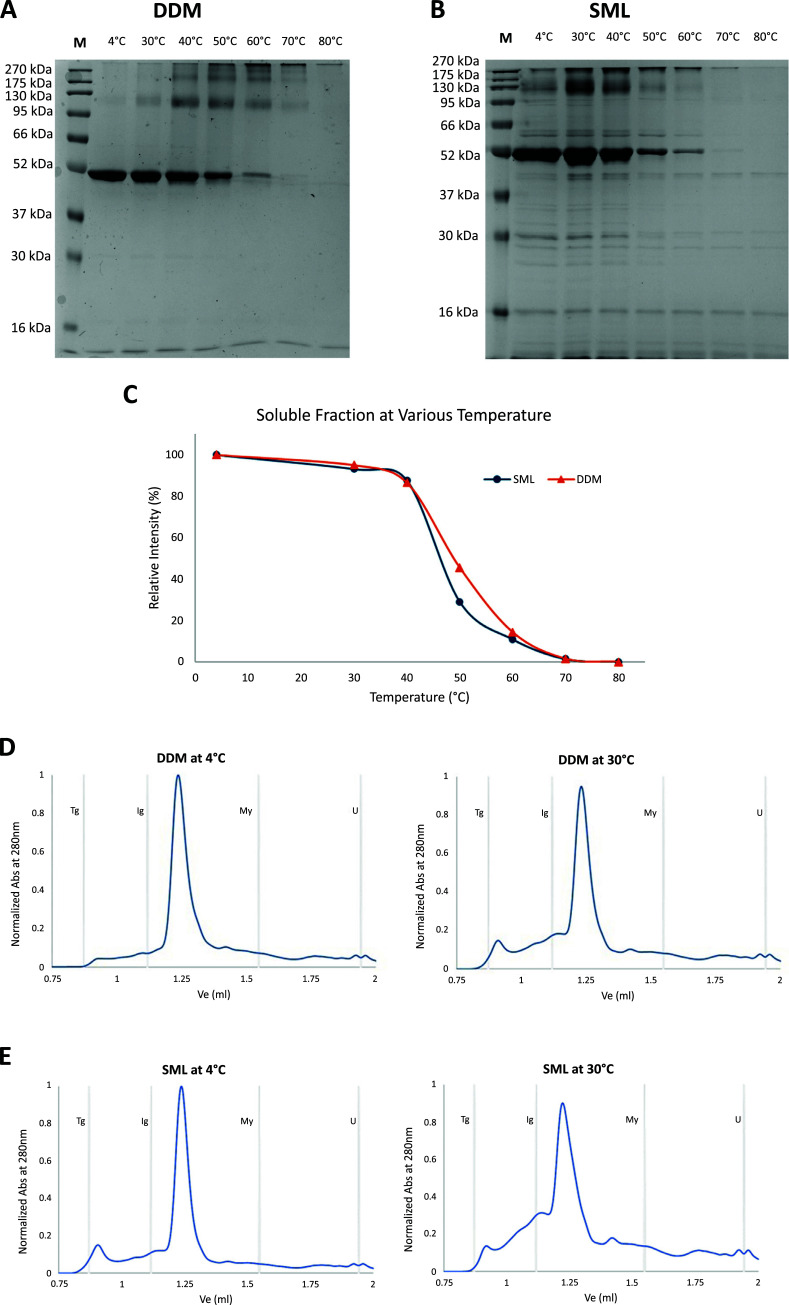
Thermostability of DDM and SML solubilized SaMurJ. SaMurJ solubilized in DDM and SML were incubated at various temperature for 30 min and the soluble supernatant were collected for (**A** and **B**) SDS-PAGE and (**D** and **E**) size exclusion analysis. (**C**) The ratio between soluble protein relative to the unincubated sample in SDS-PAGE was evaluated by ImageJ and plotted against temperature.

The thermostability of SML and DDM solubilized SaMurJ were also different. Although both samples remained soluble at 30°C and 40°C, about 70% SaMurJ was precipitated and lost in SML buffer when incubated at 50°C, whereas there was still 45% soluble protein in the DDM solubilized sample (Fig. **5A**-**C**). This indicated that SML-solubilized SaMurJ had lower stability than DDM. Size exclusion chromatography data also revealed that soluble aggregate formation in SML buffer was more profound than in DDM buffer (Fig. **5D**-**E**). Indeed, a sharp peak with a retention volume of 1.24 ml can be observed in DDM and SML samples before incubating at evaluated temperatures. For the SML sample, an additional peak eluted at 0.91 ml appeared. Upon incubation at 30°C, the SML sample had a significantly higher portion of high molecular weight species than then DDM sample. It is worth noting that even incubated at 30°C for 30 min, a significant amount of soluble aggregate was formed, which would affect the physical and biochemical characterization at or above this temperature.

### 3.4 Peptidisc Assembly

Although membrane proteins are commonly solubilized in detergent for biochemical and structural study [45-48], detergent-free environments are highly desirable in many experiments [36, 49, 50]. In this study, we tested the peptidisc system in SaMurJ with 30-, 60-, and 90-fold molar excess of NSP_r_ together with control without introducing any NSP_r_ to SaMurJ. In the control sample without NSP_r_, no SaMurJ was eluted from the nickel affinity resin, most probably due to unfolding and aggregation of the protein on the resin when it was washed by the detergent-free buffer. For samples containing 30-, 60-, and 90-folds molar excess of NSP_r_, the SaMurJ can be eluted in detergent free buffer, however, all the samples precipitated after 3 hours even stored at 4°C. We conducted the experiment again by buffer A with 5% glycerol, but precipitation still appeared (data not shown). Thus, we believed SaMurJ could transiently stabilized by NSP_r_, but the low stability would hinder the use of this system for downstream characterization.

## CONCLUSION

In this study, SaMurJ with N-terminal ten-His tag was successfully expressed in BL21(DE3) without the use of IPTG induction. One milligram SaMurJ can be obtained from 1 L culture incubated at 16°C overnight. Using a buffer containing 1% DDM to solubilize the membrane fraction of the cell pellet, SaMurJ can be extracted and further purified by nickel affinity chromatography with a purity of ~88%. The other detergent, SML could also solubilize SaMurJ but with lower purity and stability. Peptidisc also made attempts to generate detergent-free SaMurJ, but SaMurJ appears to be unstable in the absence of detergents.

## Data Availability

Not applicable.

## LIST OF ABBREVIATIONS

DDM: n-Dodecyl-β-D-maltoside
DM: n-Decyl-β-D-maltoside
HEPES: (4-(2-hydroxyethyl)-1-piperazineethanesulfonic Acid)
IPTG: Isopropyl-β-D-thiogalactoside
LDAO: Lauryldimethylamine Oxide
NSP_r_: Amphipathic bi-helical Peptide
SML: Sucrose-6-monolaurate
SDS-PAGE: Sodium Dodecyl Sulfate-Polyacrylamide Gel Electrophoresis
