# Supplementary material for: Heterogenous Expression and Purification of Lipid II Flippase from Staphylococcus aureus
Source: Protein Pept Lett. 2024 Jul 4;31(5):386–94. doi: 10.2174/0109298665316374240531113258 (PMC11348468; doi:10.2174/0109298665316374240531113258)
Supplement: Supplementary file 1 [file PPL-31-386_SD1.pdf]

Supplementary Materials

Heterogenous Expression and Purification of Lipid II Flippase from *Staphylococcus aureus*

Yuan Yuan Zheng[1], Wai-Hong Chung[1], Yun-Chung Leung[1,*] and Kwok-Yin Wong[1,*]

[1]*State Key Laboratory of Chemical Biology and Drug Discovery, Department of Applied Biology and Chemical Technology, The Hong Kong Polytechnic University, Hung Hom, Hong Kong, China*

Table S1. Detection of peptides derived from trypsin in-gel digestion of SaMurJ.

Sequence	Mass	Tgt Seq Mass	Diff (ppm)	Diff (mDa)	Base Peak
VLGVLFIIPFNYLIGGQENMAPFTYAYAPYNIAIAVATAGVPL AASKYVAKYNAIGAYK	6361.3224	6361.2629	9.35	59.47	1274.0766
NIHDKNGWSVDDITWIIRIISMVVIFIPVLATWRGIFQGYK	4813.6209	4813.5935	5.71	27.47	964.1308
HNIDRMVESDYTDIDVSYGKMYK	2854.2247	2854.2426	-6.27	-17.91	572.0522
YVAKYNAIGAYKVSQKFYK	2413.1852	2413.2067	-8.88	-21.43	805.403
IVMIPTSLSAGFAVSLIPYITK	2336.3149	2336.3065	3.56	8.31	780.1128
IVMIPTSLSAGFAVSLIPYITK	2320.3245	2320.3116	5.55	12.87	774.7834
YNAIGAYKVSQKFYK	1950.9405	1950.9588	-9.38	-18.29	651.6551
YVAKYNAIGAYKVSQK	1931.0024	1930.9901	6.36	12.27	644.6747
YNAIGAYKVSQKFYK	1864.9548	1864.9472	4.1	7.65	622.6594
MVESDYTDIDVSYGK	1736.7478	1736.7451	1.56	2.7	869.381
MVESDYTDIDVSYGK	1720.7579	1720.7502	4.5	7.74	861.3859
SMGPTAVSEVTEQIAR	1674.8284	1674.8247	2.22	3.72	838.4209
NGWSVDDITWIIR	1633.78	1633.7736	3.88	6.33	817.8971
LADEFLGEIPEKLR	1628.8857	1628.8774	5.1	8.3	543.9693
TRLADEFLGEIPEK	1616.8487	1616.841	4.76	7.7	540.2913
FKFSYSWIHFAK	1575.8016	1575.7874	8.98	14.15	788.908
LADEFLGEIPEK	1359.6954	1359.6922	2.39	3.24	680.8543
YNAIGAYKVSQK	1341.6875	1341.6929	-3.98	-5.33	671.8503
FSYSWIHFAK	1284.6328	1284.6291	2.85	3.66	429.2179
LHEMHHQIR	1215.5977	1215.5931	3.78	4.59	406.2066
LHEMHHQIR	1215.5923	1215.5931	-0.65	-0.79	406.2055
LHEMHHQIR	1199.6038	1199.5982	4.64	5.56	400.8753

0929-8665/24 © 2024 Bentham Science Publishers

Table S2. Detection of peptides derived from pepsin in-gel digestion of SaMurJ.

Sequence	Mass	Tgt Seq Mass	Diff (ppm)	Diff (mDa)	Base Peak
ITKTFAEGRLHEMHHQIRTSIGVLMFITVPASIGIM	4110.1353	4110.1217	3.32	13.66	1029.0419
NIAIAVATAGVPLAASKYVAKYNAIGAYKVSQ	3224.7293	3224.7333	-1.25	-4.04	807.4402
HHHHHHSQDAENLYFQGMSESKE	2903.1837	2903.1702	4.63	13.45	1453.0996
HHSQDAENLYFQGMSESKEMVRGT	2869.1995	2869.1919	2.64	7.56	957.7415
HHHHHHHHHHSQDAENLYFQG	2756.0398	2756.0522	-4.5	-12.41	1379.5245
HNIDRMVESDYTDIDVSYGKMY	2651.1324	2651.152	-7.37	-19.54	885.0531
SQDAENLYFQGMSESKEMVRG	2465.0653	2465.0475	7.2	17.75	1234.041
MLGVELVFFLANLFLEPTKLG	2394.2922	2394.2909	0.56	1.34	799.4397
QDAENLYFQGMSESKEMVRG	2302.0003	2301.9882	5.25	12.09	1152.5089
HNGALSLVGIPSQLQDIFFN	2169.1337	2169.1219	5.48	11.88	724.3841
VVIFIPVLATWRGIFQGY	2095.1393	2095.1506	-5.42	-11.36	1049.0783
VVIFIPVLATWRGIFQGY	2079.1656	2079.1557	4.73	9.84	1041.0914
IKLALNYPLIMLFHTPGA	2027.1339	2027.1278	3.03	6.15	677.0532
DIFFNMLNMSTNKIVM	1994.8611	1994.8675	-3.25	-6.49	998.9389
NMLNMSTNKIVMIPTS	1897.8435	1897.8471	-1.89	-3.6	950.4303
KVLGVLFIIPFNYLIG	1892.0827	1892.0812	0.82	1.55	632.0351
GRLHEMHHQIRTSIGV	1885.9684	1885.9693	-0.48	-0.91	629.9983
YYWRKRKHNIDR	1878.936	1878.935	0.51	0.96	940.9765
YPLFNLVDQFTHNGAL	1864.8986	1864.8996	-0.52	-0.96	933.9575
SVTASMLQGIDKQKLT	1849.9092	1849.9091	0.04	0.08	925.9615
KLTVYVILASVVIKLAL	1842.199	1842.1958	1.71	3.16	615.4078
HTPGAILSTSIALLFAIG	1840.0121	1840.0095	1.45	2.66	614.3451
GHDPNHDGSRLLFYY	1822.7945	1822.7911	1.89	3.45	912.4052
HNGALSLVGIPSQLQDI	1821.918	1821.9109	3.92	7.15	912.4672
VVIKLALNYPLIMLF	1762.0473	1762.0467	0.33	0.57	882.0313
VTASMLQGIDKQKLT	1734.8815	1734.8822	-0.44	-0.75	868.4479
QKFYKSSFIVMSITG	1733.8592	1733.8698	-6.12	-10.62	868.4384
FTHNGALSLVGIPSQL	1696.8918	1696.8784	7.88	13.37	1698.9018
FTYAYAPYNIAIAVA	1689.8405	1689.8403	0.14	0.24	845.9274
LHEMHHQIRTSIGV	1656.8525	1656.8518	0.42	0.69	829.4327
SLLSVTASMLQGIDK	1647.8478	1647.8502	-1.46	-2.4	824.9306
KNGWSVDDITWII	1605.7623	1605.7675	-3.22	-5.17	803.8882
FNMLNMSTNKIVM	1592.6769	1592.6772	-0.2	-0.32	797.3474
TLWYYWRKRK	1584.8347	1584.8314	2.11	3.34	793.4241
LALNYPLIMLFHT	1544.8431	1544.8425	0.41	0.63	773.4304

Sequence	Mass	Tgt Seq Mass	Diff (ppm)	Diff (mDa)	Base Peak
NKIVMIPTSLSAGF	1536.7863	1536.7858	0.33	0.5	769.4005
FAEGRLHEMHHQ	1533.6886	1533.6895	-0.63	-0.97	767.851
NLVDQFTHNGALSL	1527.7729	1527.7682	3.11	4.75	764.8938
FTYAYAPYNIAIA	1519.7268	1519.7347	-5.23	-7.94	760.8712
MSTNKIVMIPTSL	1508.7562	1508.7579	-1.1	-1.66	755.3854
AIGCNFYILKKYA	1503.7845	1503.7796	3.29	4.95	752.8996
YYWRKRKHNI	1479.7705	1479.7735	-2.02	-2.99	740.8922
YWRKRKHNID	1474.7393	1474.7429	-2.49	-3.68	738.3768
GIFQGYKSMGPTAV	1470.7192	1470.7177	1.04	1.53	736.3692
LQDIFFNMLNM	1460.6341	1460.6316	1.69	2.47	731.3246
HDGSRLLFYYAP	1453.6994	1453.699	0.28	0.4	727.8566
IGIFTLWYYW	1435.6854	1435.6812	2.88	4.14	718.8502
IAVATAGVPLAASKY	1430.8207	1430.8133	5.15	7.37	716.417
WRKRKHNID	1412.7099	1412.7021	5.46	7.71	707.3643
LIMLFHTPGAILS	1411.7926	1411.7897	2.05	2.9	706.9037
NMAPFTYAYAPY	1408.6009	1408.6009	-0.06	-0.08	705.3079
YKVSQKFYKSS	1406.7144	1406.7194	-3.57	-5.03	704.3641
FNMLNMSTNKI	1399.6114	1399.6112	0.15	0.21	700.8147
YLIGGQENMAPF	1397.6352	1397.6286	4.74	6.62	699.823
TAGVPLAASKYVAK	1374.7928	1374.7871	4.16	5.71	1375.7996
LASVVIKLALNY	1346.7722	1346.781	-6.49	-8.75	674.3931
VSYGKMYKEII	1345.6957	1345.6952	0.38	0.51	1346.7028
NYPLIMLFHTP	1345.6795	1345.674	4.03	5.42	673.8473
VIKLALNYPLI	1342.7747	1342.786	-8.46	-11.36	672.3942
FTHNGALSLVGIP	1340.7057	1340.7089	-2.33	-3.12	671.3605
IGCNFYILKK	1327.6649	1327.6595	4.08	5.42	665.3442
IAIAVATAGVPLAAS	1323.7707	1323.7762	-4.15	-5.49	662.8921
MIPTSLSAGFAVS	1322.6545	1322.654	0.38	0.5	662.3348
YKVSQKFYKS	1319.6882	1319.6874	0.59	0.78	1321.6995
AVGAIIGIFTLW	1318.7324	1318.7285	2.97	3.92	1319.7403
IIRIISMVVIF	1302.8177	1302.8098	6.09	7.93	652.4159
GKMYKEIIAYS	1301.6714	1301.669	1.89	2.46	651.8427
DGSRLLFYYAP	1300.6449	1300.6452	-0.2	-0.26	651.3299
SKYVAKYNAIG	1299.6418	1299.6459	-3.18	-4.14	1300.6485
LLSVTASMLQGI	1290.6763	1290.6853	-7	-9.04	1291.6838
IKLALNYPLIM	1287.7658	1287.7625	2.56	3.29	644.8903

Sequence	Mass	Tgt Seq Mass	Diff (ppm)	Diff (mDa)	Base Peak
ANLFLEPTKLG	1287.6839	1287.6823	1.22	1.58	644.8492
GALSLVGIPSQLQ	1283.6936	1283.6973	-2.84	-3.64	642.851
HSQDAENLYF	1281.5389	1281.5262	9.93	12.72	641.7767
TSIGVLMFITVP	1276.7097	1276.7101	-0.33	-0.42	1277.717
IYGTITIKTR	1250.6998	1250.6983	1.21	1.51	1251.7069
VFYGYDPIVL	1227.6196	1227.6176	1.66	2.04	614.8173
KSSFIVMSITG	1227.6195	1227.6169	2.06	2.53	1228.6249
KNGWSVDDIT	1220.5268	1220.531	-3.36	-4.1	1221.5333
MMLGVELVFF	1216.5976	1216.5872	8.57	10.43	1217.605
HDKNGWSVDD	1215.4779	1215.4792	-1.15	-1.39	608.7465
SESKEMVRGT	1208.5472	1208.5456	1.33	1.6	605.2809
AVSLIPYITKT	1204.7085	1204.7067	1.51	1.82	603.3612
FQGYKSMGPTA	1201.5432	1201.5438	-0.47	-0.57	601.779
ITGVLGFLVLY	1193.708	1193.706	1.68	2	1194.7157
YLIGGQENMAP	1191.566	1191.5594	5.53	6.59	1192.5732
NAIGAYKVSQK	1178.6249	1178.6295	-3.92	-4.62	1179.6321
ANGIATFAAAVGA	1175.5857	1175.5935	-6.67	-7.84	1176.5925
KHNIDRMVE	1173.5565	1173.5448	9.92	11.64	1174.5641
KSSFIVMSIT	1170.6007	1170.5955	4.48	5.25	1171.6076
SYSWIHFAK	1169.5491	1169.5506	-1.26	-1.48	1170.5573
IGGQENMAPFT	1163.5305	1163.5281	2.02	2.35	1164.5387
GIPSQLQDIF	1161.5576	1161.5554	1.94	2.25	1162.5648
MYKEIIAYS	1159.5553	1159.5584	-2.6	-3.01	1160.5623
TRLADEFLGE	1149.5673	1149.5666	0.61	0.7	1150.5746
YKVSQKFY	1148.5531	1148.5502	2.52	2.9	1149.5601
FITVPASIGIM	1147.6344	1147.6311	2.89	3.32	1148.6421
HNGALSLVGIP	1136.5809	1136.5826	-1.5	-1.7	1137.5884
ANGIATFAAAVGA	1132.5802	1132.5877	-6.56	-7.43	1133.5872
TLWYYWR	1129.5364	1129.5345	1.69	1.91	1130.5436
RKRKHNID	1125.6046	1125.6003	3.82	4.3	1126.6123
PIVLGHDPNH	1114.539	1114.5407	-1.58	-1.76	1115.5463
MLNMSTNKI	1110.5045	1110.5049	-0.4	-0.44	1111.512
YFLAPYISE	1101.5386	1101.5383	0.3	0.33	1102.5458
NIAIAVATAGVP	1095.6233	1095.6288	-5.06	-5.54	1096.6303
GSRLLFYYA	1088.5704	1088.5655	4.54	4.94	1089.5774
TEQIARVIF	1076.5892	1076.5866	2.43	2.62	1077.5969

Sequence	Mass	Tgt Seq Mass	Diff (ppm)	Diff (mDa)	Base Peak
SEVTEQIAR	1074.5276	1074.5306	-2.76	-2.97	1075.5349
LIGGQENMAP	1073.4731	1073.4699	2.94	3.16	1074.4801
SYLVLNVFD	1069.5333	1069.5332	0.11	0.11	1070.5406
SHHHHHHH	1064.4523	1064.455	-2.48	-2.64	1065.4599
QPLFTVFY	1057.513	1057.5121	0.9	0.95	1058.5197
FYYAPVAIL	1055.5716	1055.5692	2.32	2.45	1056.5794
IAVATAGVPLAA	1052.6271	1052.623	3.89	4.09	1053.6342
KNGWSVDDI	1048.4737	1048.4825	-8.42	-8.83	1049.4814
FLGEIPEKL	1044.5868	1044.5855	1.19	1.24	1045.5938
YAYAPYNIA	1044.4947	1044.4916	2.93	3.06	1045.5022
YKSMGPTAVS	1039.5012	1039.5008	0.38	0.39	1040.5082
MLQGIDKQ	1034.4609	1034.4703	-9.02	-9.33	1035.4681
FIIPFNYL	1025.5627	1025.5586	4.01	4.12	1026.5698
YSIPFVIVS	1023.5665	1023.5641	2.31	2.37	1024.5733
RGTFLITIS	1006.5843	1006.5811	3.17	3.19	1007.5917
ITVPASIGIM	1000.5631	1000.5627	0.38	0.38	1001.5704
SIGIMALAQP	1000.5238	1000.5263	-2.48	-2.48	1001.5307

Table S3. Physical properties of detergent used in solubilization of SaMurJ.

Full Name	Abbreviation	M.W. (g/mol)[1]	CMC (mM)[1]	HLB	Micelle M.W.
Brij L23	BJ	1198.00	0.091	16.9[1]	48,000[1]
n-Dodecyl-β-D-maltoside	DDM	510.62	0.17	13.4[2]	40,000 – 76,000[1]
Sucrose monolaurate	SML	524.60	0.30	13.0[2]	18,500 – 51,900[3]
n-Decyl-β-D-maltoside	DM	482.56	1.80	14.1[2]	33,300[1]
n-Octyl- β-D-glucoside	OG	292.37	18 – 20	12.3[2]	7,900 – 29,200[1]
Lauryldimethylamine oxide	LDAO	229.40	1 – 2	5.2[2]	17,400[1]

Noted: [1]*A guide to the properties and uses of detergents in biological systems*, Sigma-Aldrich [1]
[2]Calculate according to Griffin's equation HLB = 20 × M_h / M_w where M_h is the molecular mass of the hydrophilic moiety, and M_w is the molecular mass of the whole molecule [2].
[3]Work from Susana A et al [3]

REFERENCES

[1] Bhairi, S.M.M.C.; Ibryamova, S.; LaFavor, T. *A guide to the properties and uses of detergents in biological systems*. Available from: https://www.sigmaaldrich.com/deepweb/assets/sigmaaldrich/marketing/global/documents/267/427/detergents-guide-mk.pdf.

[2] Griffin, W.C. *Classification of surface-active agents by "HLB"*. Journal of Cosmetic Chemists, 1949. **1**(5): p. 8-23.

[3] Sanchez, S.A. *et al., Sucrose monoester micelles size determined by Fluorescence Correlation Spectroscopy (FCS)*. PLoS One, 2011. **6**(12): p. e29278.
